# Effects of Preparation Procedures and Porosity on Thermoelectric Bulk Samples of Cu_2_SnS_3_ (CTS)

**DOI:** 10.3390/ma15030712

**Published:** 2022-01-18

**Authors:** Ketan Lohani, Carlo Fanciulli, Paolo Scardi

**Affiliations:** 1 Department of Civil, Environmental & Mechanical Engineering, University of Trento, Via Mesiano 77, 38123 Trento, Italy; ketan.lohani@unitn.it; 2 Lecco Unit, National Research Council of Italy–Institute of Condensed Matter Chemistry and Technologies for Energy (CNR-ICMATE), Via Previati 1/E, 23900 Lecco, Italy; carlo.fanciulli@cnr.it

**Keywords:** copper tin sulfide, Cu_2_SnS_3_, CTS, thermal stability, chalcogenide, material production, porosity, porous thermoelectric materials

## Abstract

The thermoelectric behavior and stability of Cu_2_SnS_3_ (CTS) has been investigated in relation to different preparations and sintering conditions, leading to different microstructures and porosities. The studied system is CTS in its cubic polymorph, produced in powder form via a bottom-up approach based on high-energy reactive milling. The as-milled powder was sintered in two batches with different synthesis conditions to produce bulk CTS samples: manual cold pressing followed by traditional sintering (TS), or open die pressing (ODP). Despite the significant differences in densities, ~75% and ~90% of the theoretical density for TS and ODP, respectively, we observed no significant difference in electrical transport. The stable, best performing TS samples reached *zT* ~0.45, above 700 K, whereas *zT* reached ~0.34 for the best performing ODP in the same conditions. The higher *zT* of the TS sintered sample is due to the ultra-low thermal conductivity (*κ* ~0.3–0.2 W/mK), three-fold lower than ODP in the entire measured temperature range. The effect of porosity and production conditions on the transport properties is highlighted, which could pave the way to produce high-performing TE materials.

## 1. Introduction

Thermoelectric (TE) materials are a class of functional materials employed in solid-state energy generators and coolers, without the use of fossil fuels and combustion processes, and with no moving parts, exploiting only the temperature gradients. For this reason, TE materials were first successfully utilized in deep-space probes in the second half of the last century [1]. In recent years, TE materials have drawn attention as efficient, eco-friendly, durable, noise-free, and scalable solutions for the recovery of waste heat, like in exhaust systems (automotive TE generators) [2]. Current research on TE materials seeks greater efficiency, as well as non-toxic and cost-effective solutions [3]. The performance of a TE material can be assessed by means of the figure of merit, *zT* = (*S*^2^/*ρκ*)*T*, where *S*, *ρ*, *κ*, and *T* are the Seebeck coefficient, electrical resistivity, thermal conductivity, and absolute temperature, respectively. Thus, a high *zT* TE material requires a high power factor (*S*^2^/*ρ*) and low *κ*, which consists of lattice (*κ_l_*) and electronic (*κ_e_*) contributions. That being said, it is not the only important parameter as the stability and durability of TE materials are also vital for producing reliable TE devices.

Cu-Sn-S-based systems are studied as both *p*-type and *n*-type semiconductors [4,5]. CTS is a tetrahedrally bonded direct bandgap semiconductor, showing a bandgap ~1.0 eV, which is higher or lower depending on whether the CTS polymorph is ordered or disordered [6]. The unfilled Cu-*3d* orbitals introduce holes in the CTS system, making it a *p*-type semiconductor. CTS components are earth-abundant, eco-friendly, non-toxic, non-hazardous, and have low formation energies; therefore, they are explored for various applications, such as TE [7], photovoltaic [8], optoelectronics [9], photoelectrochemical [10], and supercapacitors [11], just to name a few. CTS has attracted the TE community for its remarkable crystallographic structures with different degrees of cation-disorder, which helps in the suppression of thermal conductivity to an ultra-low level, without much hindrance to the electronic transport, also known as the so-called “phonon-glass-electron-crystal (PGEC)” behavior. The well-known ordered or monoclinic CTS polymorph arranges its atoms in the *Cc* space group (SG). In the past, we reported the production of a pure disordered or cubic CTS polymorph (SG: *F-43m*) via high-energy reactive ball-milling [4]. Recently, Wei et al. [12] reported the production of another disordered CTS polymorph, i.e., pure tetragonal (SG: *I-42m*) CTS by the colloidal method.

Interestingly, both the disordered CTS polymorphs (cubic and tetragonal) present *κ_l_* <0.5 W/m-K, above 700 K, approaching its theoretical minimum (*κ_l_* ~0.3 W/m-K). In a recent report, Li et al. [13] showed the introduction of dislocations as an effective way to suppress the lattice thermal conductivity in Ni-doped CTS (Cu_2_Ni_0.1_Sn_0.9_S_3_), reporting *κ_l_* = 0.41 W/m-K at 723 K. Wei et al. [12] demonstrated that the abundance of twin-boundaries in pristine and In-doped CTS (Cu_2_Sn_0.95_In_0.05_S_3_) contributes to a low *κ_l_* = 1.02 W/m-K at room temperature (RT), which further reduces to *κ_l_* < 0.50 W/m-K around 700 K. It is important to notice that most materials presenting a high *zT* in the medium temperature range (500 K < *T* < 800 K) are either scarce or toxic, e.g., PbTe and SnSe. In this temperature range, sulphides of copper and tin or copper and iron can be viable alternatives. Gu et al. [14] discussed 3D modulation doping of CuCo_2_S_4_ in CTS and reported a *zT* ~0.82 at 773 K. Zhao et al. [15] reported simultaneous Co and Sb substitution in the CTS system resulting in a *zT* of ~0.88 at 773 K. Furthermore, Deng et al. [16] reported a *zT* of ~1 in a similar Cu_7_Sn_3_S_10_ system through Br-doping. A *zT* of ~0.4–0.8 above 700 K is regularly reported for CTS by In [17], Zn [7], Mn [18], Ni [19], Fe [20], Co [21], and Cu [22] substitution at the Sn site, aiming to enhance the carrier concentration and reduce the thermal conductivity. Other similar systems, such as Cu_5_Sn_2_S_7_ [23], CuFeS_2_ [24], Cu_4_Sn_7_S_16_ [25], and Cu_2_SnSe_3_ [26] also show a moderate *zT* ranging from 0.2–0.5, above 700 K.

Manipulation of porosity in materials is a less-explored subject for TE applications. Many articles have put forward a crucial need to investigate porous TE materials [3,27,28,29]. Porous materials are lightweight and require lower quantities of materials, therefore, they are suitable for portable and wearable TE devices [27]. Xu et al. [30] showed *zT* > 1, and 2–4 fold lower *κ_l_* in hollow nanostructured Be_2_Te_2.5_Se_0.5_ than in the existing literature. Hong et al. [31] showed improved PF in mesoporous ZnO thin films. Tiwari et al. [32] investigated porous CTS for photovoltaics using a solid-state reaction at 200 °C, but they only reported the Seebeck coefficient near RT and temperature-dependent conductivity measurement. Thermal cycling during the TE power generation process could promote the annealing of defects, reduction of grain boundaries, increased density, and transformation of crystal structures, driving these materials towards instability. The authors discussed surface oxidation, the role of secondary phases, and the loss of a nano and microstructure after thermal cycling above a certain temperature, which alters the TE properties of a material [33]. This makes the technological advancement and scientific understanding of these materials challenging [34]. In particular for large-scale production and durable use, a mechanically and thermodynamically stable material is required.

Herein, we stabilized the disordered CTS polymorph using different production conditions and sintering techniques. CTS samples from two batches (three samples each) were produced, and their TE properties and thermal stability were systematically investigated by repeated Seebeck and resistivity measurements (heating and cooling) in the temperature range of 323–723 K. Pellets of samples were produced using manual cold pressing followed by sintering in a traditional tubular furnace (TS batch), or via open die pressing (ODP batch) [35]. Information on the crystallographic structures and phase purity was obtained by Rietveld refinement of X-ray diffraction (XRD) patterns, before and after the repeated TE measurements. Surface topology, porosity, and chemical variations were investigated by morphological images and energy dispersive X-ray (EDX) spectroscopy, using both scanning electron microscopy (SEM) and transmission electron microscopy (TEM). This work provides a general overview and highlights the cautions to produce stable CTS and similar chalcogenides. The effect of porosity on TE properties is highlighted.

## 2. Materials and Methods

CTS samples were produced from the elemental powders using a high-energy reactive ball-mill (Fritsch, Idar-Oberstein, Germany), according to a well-assessed procedure with details discussed elsewhere [4]. Three different samples were prepared using the manual cold press (load 15 tons for 3 min) and sintered at 500 °C for 2 h with a heating rate of 3 °C/min. Sample TS1 was processed in an open environment, whereas samples TS2 and TS3 were produced in a highly controlled environment (O_2_ and H_2_O < 10 ppm). All the samples were sintered in Ar flux. Additionally, sample TS3 was sintered in the presence of sulfur powder (1 g).

The other three samples (ODP1, ODP2, and ODP3) were produced in a controlled environment with the above-mentioned preconditions. Differently, the sintering was performed via ODP using an iron die. To prevent possible reactions between the die and the powders, a boron nitride layer was used to cover the internal surface of the die. Such a layer also prevents sticking phenomena, allowing for an easier extraction of the sintered bulk. Sample ODP1 was sintered at 400 °C for 30 min, whereas samples ODP2 and ODP3 were sintered at 500 °C for 10 and 30 min, respectively. During all the sintering processes, a ~100 MPa axial pressure was applied.

It should be emphasized that ODP was performed on a large quantity of the as-milled powder, namely ~30–40 g. The as-sintered samples from ODP are usually large bulk samples, which are cut into the required sample sizes for various measurements (large as-sintered ODP samples are shown in Figure S1). The densities of the TS (TS1, TS2, and TS3) and ODP (ODP1, ODP2, and ODP3) sintered samples were ~75% and ~90% of the theoretical density (see Table 1), respectively.

The structural characterization of TS samples was performed in Bragg–Brentano geometry using a Rigaku PMG (Rigaku PMG, Tokyo, Japan) powder diffractometer, equipped with a Cu *Kα* (*λ* = 1.5406 Å) source and a scintillation counter detector. XRD data on the ODP samples were collected in Bragg–Brentano geometry using a Bruker D8 (Bruker, Billerica, MA, USA) diffractometer equipped with a Co *Kα* (*λ* = 1.7889 Å) source and area linear (1D) detector. After the phase identification, Rietveld refinement [36] was performed using Whole Powder Pattern Modelling (WPPM) macros [37], as implemented in Topas 7 [38] software (Coelho Software, Brisbane, Australia).

Simultaneous Seebeck coefficient and resistivity measurements were performed in four contact set-up using LSR-3 (Linseis Messgeraete GmbH, Selb, Germany), at a temperature ranging from 323–723 K. Thermal diffusivity (D) was measured using a LFA-500 (Linseis Messgeraete GmbH, Selb, Germany), equipped with a xenon-flash lamp over a temperature range of 323–723 K. Seebeck and resistivity measurements were performed under an He atmosphere (0.1 bar). However, the diffusivity was measured under a vacuum (~10^−3^ bar).

After the TE measurements, morphological images of the selected area electron diffraction (SAED) were collected on powder produced by crushing the sample pellets, and EDX spectroscopy was performed using transmission electron microscopy (TEM; HR-S/TEM ThermoFischer TALOS 200 s, Thermo Fischer Scientific, Waltham, MA, USA). Moreover, morphological images and EDX data were collected on pellet samples to investigate the microstructure and bulk chemistry using a Jeol IT300 scanning electron microscope (SEM) equipped with a tungsten source (Jeol, Ltd., Tokyo, Japan).

## 3. Results and Discussions

**Figure 1:**
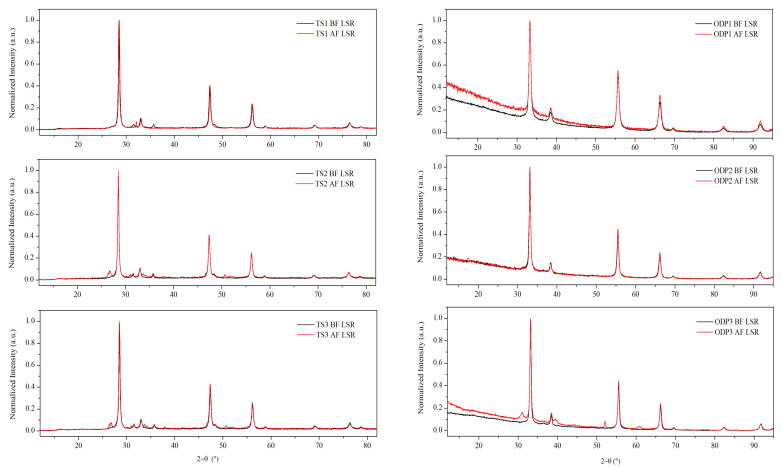
XRD data before and after repeated TE measurements on TS and ODP sintered samples. The difference in diffuse background and shifts in Bragg peaks between TS and ODP samples is due to the use of different diffractometers equipped with Cu *Kα* (*λ* = 1.5406 Å) and Co *Kα* (*λ* = 1.7889 Å) sources, respectively. Rietveld refinement on the XRD data with marked phases is shown in Figure S2.

XRD measurements were performed on all of the polycrystalline samples (Figure 1). The diffraction patterns showed fingerprint peaks at *2θ*~28.5°, 47.3°, and 56.0°, and *2θ* ~33.2°, 55.5°, and 66.3° using Cu *Kα* and Co *Kα* X-ray sources, respectively. These Bragg peaks represent planes (1 1 1), (2 2 0), and (3 1 1), respectively, confirming the disordered cubic CTS structure, which is derived from the zinc-blende structure (SG: *F*-43*m*). A small amount of WC (SG: *P-6m2*) is present in all of the samples due to the use of WC vials for milling. Additionally, a weak peak (*2θ* ~27°) of SnO_2_ (SG: *P*_42_*mnm*) was observed in the pattern of sample TS1 (see Figure S2). XRD patterns of all the ODP sintered samples also showed Bragg peaks representing the disordered cubic CTS phase. Nevertheless, small amounts of WC and SnO/SnO_2_ phases were also observed in some ODP sintered samples.

Different crystallographic phases were identified, and their respective weight fractions were obtained by the Rietveld method [36,39]. Information provided by powder pattern refinements, including the average crystallite size, is reported in Table 1. The lattice parameters for all the samples are *a* = 5.43 ± 0.01 Å and *α* = *β* = *γ* = 90°. The average crystallite size for samples TS1, TS2, and TS3 is 40 ± 10, 75 ± 10, and 70 ± 10 nm, respectively. All the samples have a ~1–2% weight of WC. Additionally, sample TS1 has a small quantity (~2% weight) of SnO_2_, which is possibly formed due to open environment processing. We did not observe the formation of secondary phase oxides in samples TS2 and TS3, produced in a strictly controlled environment. ODP, like spark plasma sintering (SPS), is a fast-sintering technique needing a short sintering time and applying external pressure. The applied pressure during ODP decreases the sintering temperature and time, simultaneously improving the densification kinetics. Thus, the crystalline domains show limited growth, while reaching full densification. The average domain sizes for the ODP1, ODP2, and ODP3 samples are 42 ± 10, 54 ± 10, and 64 ± 10 nm, respectively. In our recent work, we showed that SPS on as-milled CTS powder can constrain the grain growth below 50 nm [40].

After the repeated TE measurements (discussed in the next section), the XRD data were collected for the second time. All TS samples showed an increase in the weight fraction of SnO_2_. Samples TS2 and TS3 showed the formation of a new secondary phase, SnO (SG: *Pnm*_21_), possibly due to an oxygen-deficient production environment. We also observed the presence of SnS (SG: *Pbmn*) in sample TS1, confirming the chemical instability of the sample prepared in an open environment. Samples sintered using ODP were less prone to growth in the secondary phases. We did not observe any grain growth or increase in the weight fraction of secondary phases for ODP1 and ODP2. The applied pressure during sintering seems to better stabilize the samples than traditional sintering. Nevertheless, an increased amount of SnO_2_ was observed for ODP3, due to its extended sintering time in comparison to ODP2. The densities of ODP3 and ODP2 samples are similar, in spite of the different sintering times of 30 and 10 min, respectively. Therefore, the densification was complete within ~10 min of sintering. Furthermore, extended sintering mainly promotes phase degradation, leading to the formation of SnO_2_.

As discussed in the Introduction, the loss of nanostructure has adverse effects on the TE properties of the material, which could drive it towards instability. The average crystalline size for the TS1 sample increased from 40 ± 10 to 75 ± 10 nm after repeated TE measurements, whereas samples TS2 and TS3 did not lose their nanostructure, maintaining their average crystallite size of 75 ± 10 and 70 ± 10 nm, respectively. Similarly, the ODP sintered samples maintained their average domain size of 42 ± 10, 54 ± 10, and 68 ± 10 nm for ODP1, ODP2, and ODP3 samples, respectively, after repeated TE measurements.

**Figure 2:**
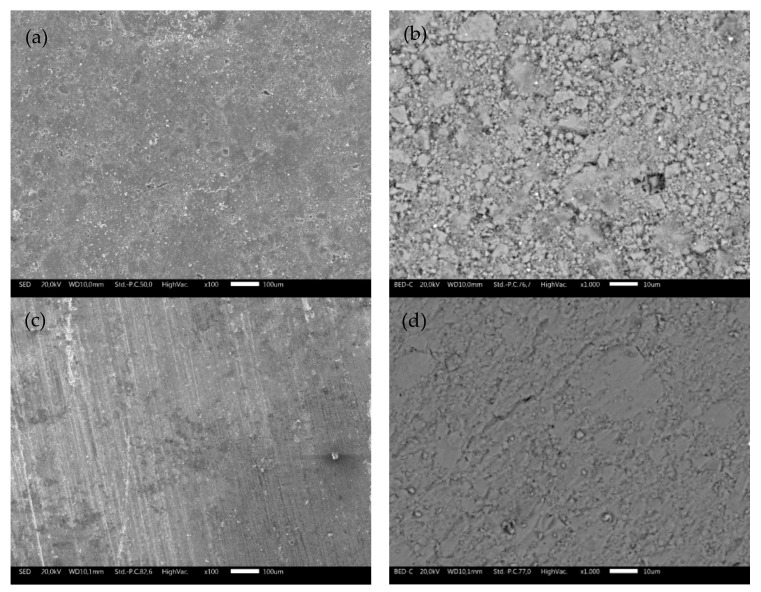
SEM micrographs collected on the surfaces of bulk TS (**a**,**b**) and ODP (**c**,**d**) sintered samples showing highly intact grains with a lower porosity in comparison with the TS sintered samples at different magnifications.

SEM micrographs at different magnifications collected on TS and ODP sintered sample surfaces are shown in Figure 2. The difference between the two samples is noticeable. Morphological images of the bulk TS samples (Figure 2a,b) reveal an uneven topology and the presence of microscopic pores. As expected, the ODP sintered samples (Figure 2c,d) had a lower porosity compared to TS, with intact grains. It is also evident through the density measurements that the ODP sintered samples were ~20% denser than TS. Here, it is worth mentioning that similar samples sintered via SPS showed almost no porosity and the density was slightly (~2–4%) higher than the ODP sintered samples (morphological images of SPS sintered samples are shown in Figure S3) [40].

The morphological image and SAED using TEM on TS1, shown in Figure S4 (right bottom inset), reveals many small (d~10 nm) grains surrounding the bigger (CTS) grains. EDX was performed, focusing on the surrounding small dark grains. Strong peaks of oxygen and tin can be observed in the EDX spectra of TS1, and the atomic fraction was found to be oxygen and tin-rich (see Table 2), suggesting that these are SnO_2_ grains, as we observed from the XRD. The evolution of SnO_2_ grains makes the sample unstable during TE measurements by unbalancing the stoichiometry. For samples TS2 and TS3, no SnO_2_/SnO grains appeared in the micrographs, although a weak peak for oxygen could be observed by EDX. The chemical composition for the TS2 sample was copper-rich, whereas, for the sulfurized sample (TS3), the stoichiometry was close to the theoretical (see Table 2).

**Figure 3:**
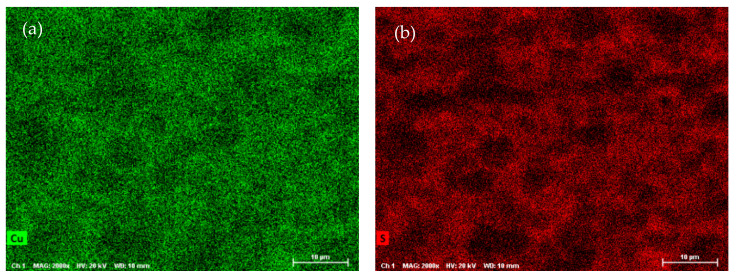

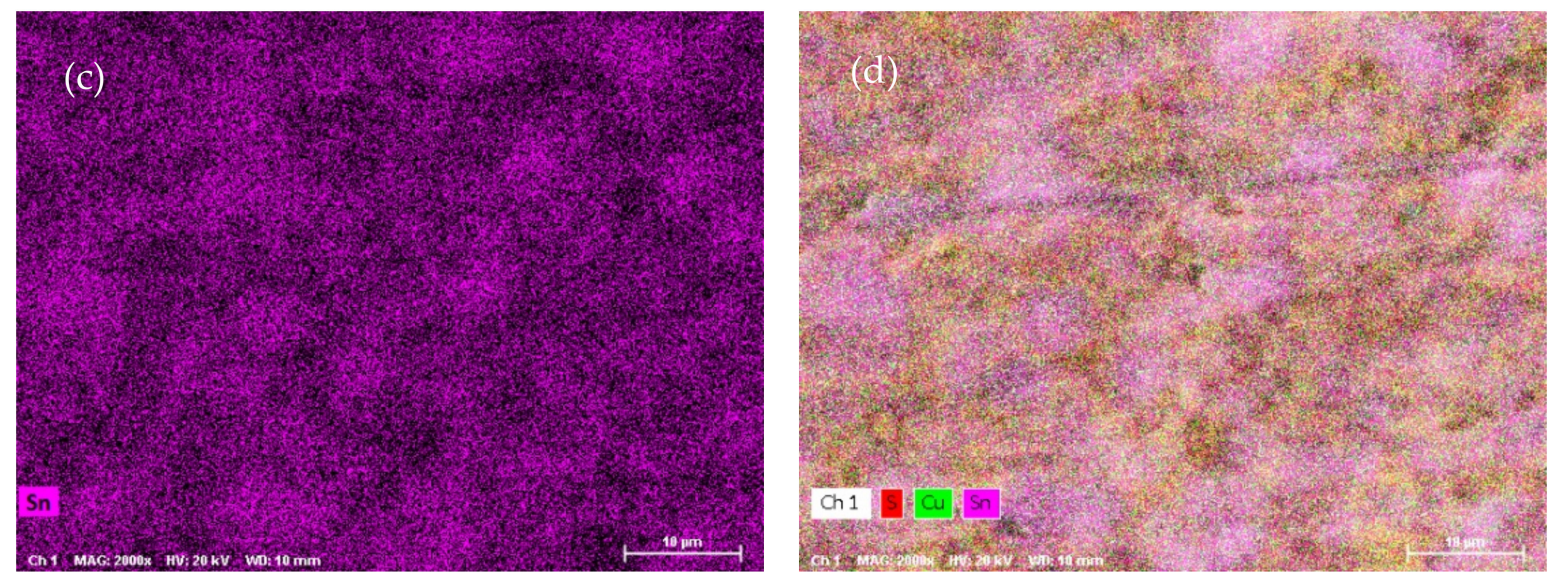
SEM micrographs collected on ODP samples and chemical maps for individual elements Cu (**a**), S (**b**), and Sn (**c**), and simultaneously for all elements (**d**) (corresponding EDX spectra are shown in Figure S5).

SEM-EDX analysis was performed on the ODP sintered samples, including chemical maps on large ODP samples to investigate the overall chemical homogeneity. As discussed in the Experimental Methods, ODP was performed on a large quantity (30–40 g) of ball-milled powder, producing large samples (see Figure S1). The SEM chemical maps (shown in Figure 3) revealed non-homogeneous chemistry on the surfaces of the ODP sintered samples. As ODP is a fast-sintering technique and was performed on a large quantity of materials, variations in chemical composition are likely. However, the overall chemistry of the samples was close to theoretical (shown in Table 3). On the contrary, the SPS sintered samples produced in our past work showed a homogeneous chemical distribution and comparatively lower crystalline domain size [40].

**Figure 4:**
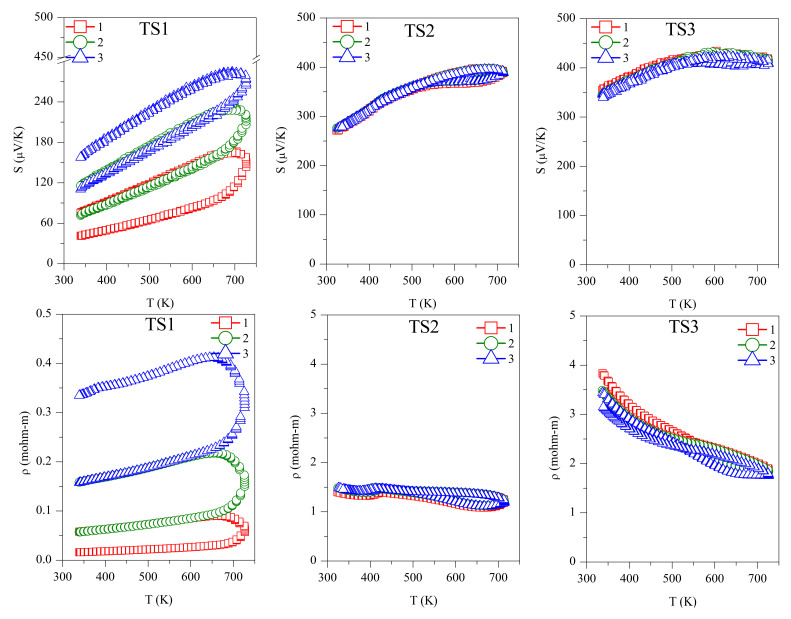

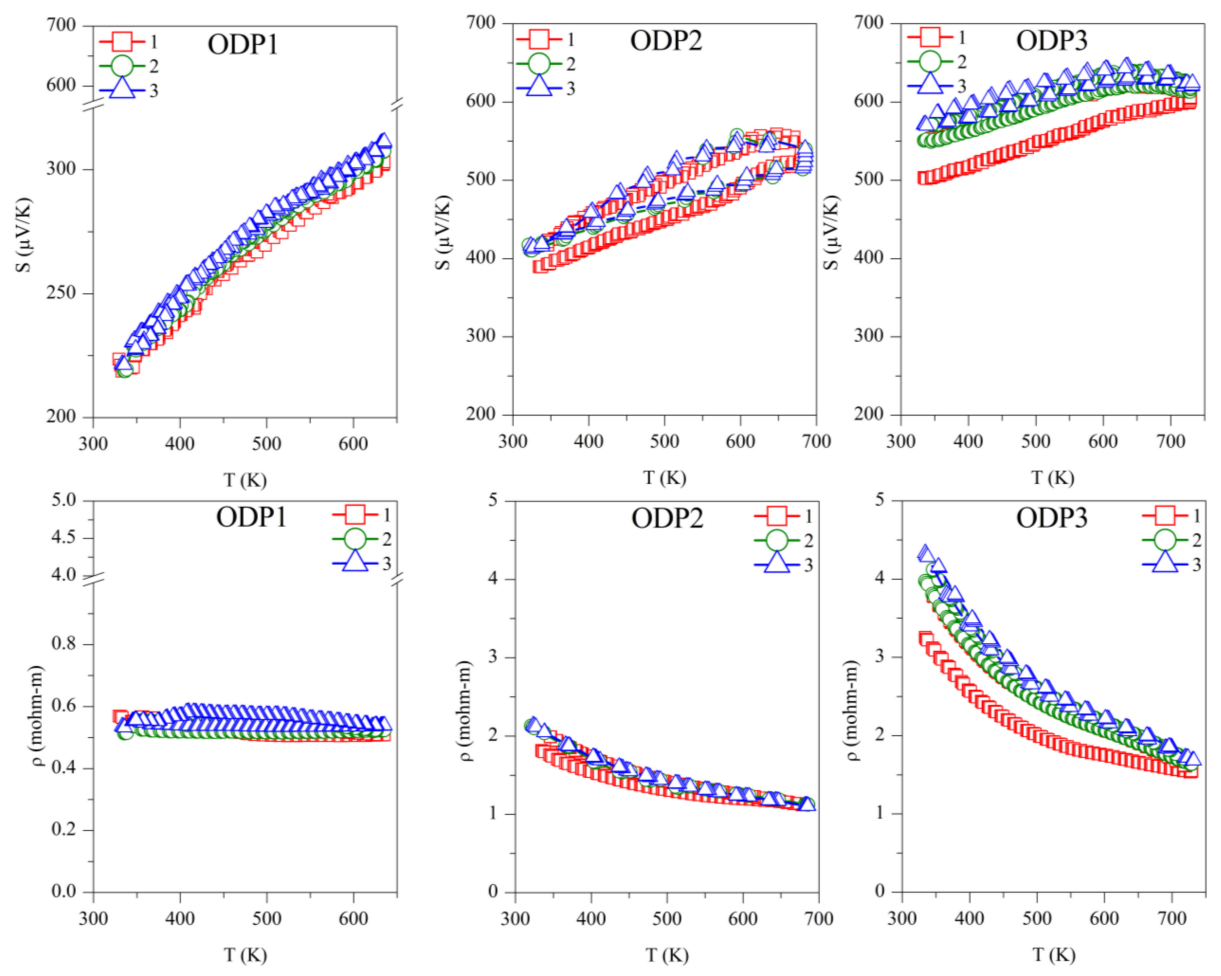
Repeated absolute Seebeck coefficient (*S*) and resistivity (*ρ*) measurements in temperature for the TS and ODP sintered samples. In the figure, markers 1 (red squares), 2 (green circles), and 3 (blue triangles) represent first, second, and third measurements cycles, respectively.

Repeated Seebeck and resistivity measurements are shown in Figure 4. All the samples, and TS and ODP batches, showed positive values for the absolute Seebeck coefficient, indicating holes as majority charge carriers, thus confirming a *p*-type semiconducting nature. The trend of resistivity for sample TS1 is increasing with the temperature, whereas it is decreasing for all other samples, typical of degenerate and non-degenerate semiconductors, respectively.

The first measurement on the TS1 sample shows the lowest values of *S* (40–100 µV/K) and *ρ* (0.01–0.03 mΩ-m). However, these measurements are not reproducible during the second and third measurement cycles. The low Seebeck and resistivity with a non-degenerate semiconducting trend suggest the presence of a higher carrier concentration in sample TS1. This could be due to the alteration in chemical composition, as we observed a large amount of SnO_2_ using TEM-EDX and XRD. The XRD on the same sample after repeated TE measurement cycles revealed the segregation of SnS (see Table 1), making the sample copper-rich or possibly leading to the formation of CuS. In a recent work on non-stoichiometric (copper-rich) CTS [18], lower Seebeck (~50 µV/K at 300 K) and high electrical conductivity were reported. Other than the formation of SnS and SnO_2_, the non-repeatability of *S* and *ρ* during the thermal cycles also confirms the instability of sample TS1. Noticeably, after the three measurements cycles, significant grain growth was observed for TS1, with the average domain size increasing from 40 ± 10 to 75 ± 10 nm.

For samples TS2 (*S* ~250–400 µV/K, *ρ* ~1.5–1.1 mΩ-m) and TS3 (*S* ~325–425 µV/K, *ρ* ~3.5–1.5 mΩ-m), the TE measurements were repeatable during the measurement cycles, and the values of *S* and *ρ* were in agreement with the literature [7,15]. Although we observed an increased amount of SnO_2_ and the formation of a new SnO phase, segregation of SnS was not observed. Similar values of *S* and *ρ* during several measurement cycles confirmed that the CTS samples prepared in a strictly controlled environment were stable. It is known that sulfurization leads to the formation of stoichiometric CTS [41], which could be the case for sample TS3, leading to a higher Seebeck and resistivity.

The repeated TE measurements on ODP sintered samples were reproducible, especially after the first measurement cycle, and for the second and third measurements cycles, the *S* and *ρ* measurements were coherent. The ODP1 sample sintered at 400 °C for 30 min showed the lowest values of *S* (180–310 µV/K) and *ρ* (0.55–0.50 mΩ-m). ODP2 and ODP3 samples sintered at 500 °C for 10 min and 30 min showed increasing values of *S* and *ρ*. The Seebeck coefficients and resistivities for the ODP2 and ODP3 samples were *S* ~400–550 µV/K and *ρ* ~2.2–1.0 mΩ-mm, and *S* ~550–650 µV/K and *ρ* ~4.0–1.7 mΩ-m, respectively. The increase in *S* and *ρ* have a clear relation with the increased average domain size of the samples. These results are in agreement with our recent work showing the effects of grain growth on SPS sintered CTS samples, owing to the conduction based on the metallic nature of surfaces due to dangling bonds [40]. Moreover, Ming et al. [42] showed a similar effect in Cu_2_SnSe_3_.

However, it is important to notice here that two different sintering were used for the sample preparation, resulting in significantly different densities, even if we did not observe a significant difference in the resistivity of the same samples. Thus, we put forward that electrical transport is less likely affected by porosity in these systems. However, we noticed that the TS sintered samples had a lower *S* in comparison with the ODP samples. This could be related to the overall chemical composition of the TS samples, where we noticed a large chemical fluctuation in TEM-EDX. Some chemical fluctuations were also present in the ODP samples, but the overall chemistry of ODP samples was close to the theoretical, as observed by means of the chemical maps.

Thermal diffusivity (*D*) was measured using a xenon flash instrument, and the thermal conductivity (*κ*) was calculated as, *κ* = *D* × *d* × *C_p_*, where, *d* and *C_p_* are the density of the sample and specific heat capacity, respectively. The density was measured using the Archimedes method (see Table 1), and *C_p_* obtained on TS samples is discussed elsewhere [4], whereas *C_p_* measurements on ODP sintered samples are shown in Figure S7.

**Figure 5:**
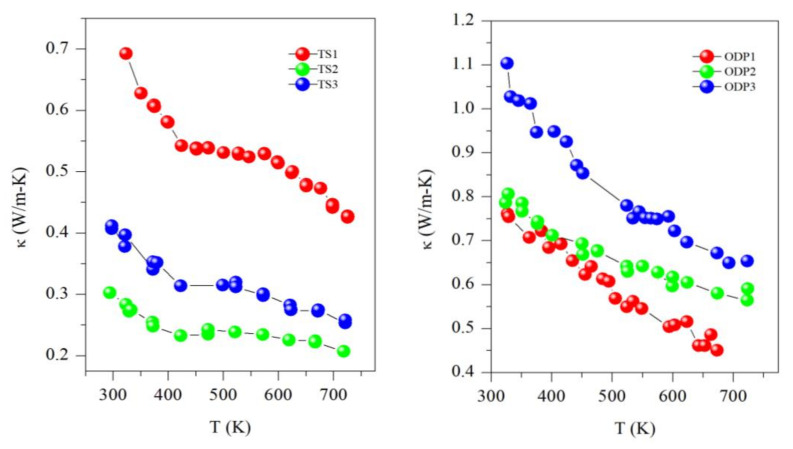
Thermal conductivity (*κ*) for TS and ODP sintered samples.

All the samples showed a decreasing trend of thermal conductivity with temperature, implying that the phonon–phonon (Umkalpp process) is dominating the phonon transport (see Figure 5). Here, it is worth mentioning that all the samples of this work have a disordered cubic structure with partial occupancies of cations (Cu and Sn), which results in a comparatively lower thermal conductivity than the ordered or monoclinic CTS polymorph [6]. The thermal conductivity of sample TS1 ranges from 0.70 W/mK to 0.45 W/mK, whereas it is ultra-low for samples TS2 and TS3, with values of 0.40–0.30 W/mK and 0.30–0.20 W/mK, respectively, in the entire measured temperature range of 323–723 K. Samples TS2 and TS3 have a similar *κ* as we recently reported for CTS [4]. However, the unstable sample TS1 has the highest *κ* in the TS batch. The higher thermal conductivity of sample TS1 is due to its higher electrical conductivity, as observed by the degenerate semiconductor trend of resistivity, probably resulting from the formation of Cu-rich CTS or CuS. The thermal conductivity of ODP sintered samples is in the range of other CTS and Cu-Sn-S-based systems [7,18]. A clear correlation between densities and *κ* can be noticed in the ODP samples as well. The densities of ODP samples are similar to the literature for CTS polymorphs [7,12,18]. The difference in *κ* between TS2 and TS3 is also likely to be due to their lower and higher densities, respectively. It is noteworthy that the *κ* of TS samples is ~3-fold lower in the entire temperature range than for the ODP sintered samples. However, we observed a more or less same behavior for the electrical transport, e.g., samples TS2 and ODP2 have *ρ*~1.5–1.0 mΩ-m and ~2.0–1.2 mΩ-m, respectively, but TS2 has a ~3-fold lower *κ* (0.3–0.2 W/mK) than ODP2 (0.8–0.6 W/mK).

**Figure 6:**
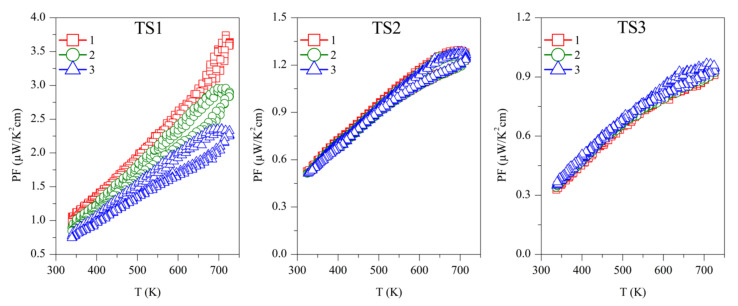

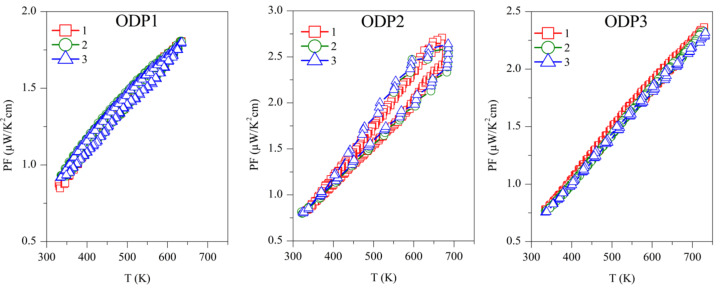
Repeated power factor (PF) for TS and ODP sintered samples. In the figure, markers 1 (red squares), 2 (green circles), and 3 (blue triangles) represent the power factors during the first, second, and third measurements cycles, respectively.

The power factor was calculated as *PF* = *S^2^*/*ρ*, and is shown in Figure 6. The first measurement on Sample TS1 has the highest *PF* ~3.5 µW/K^2^ cm, above 700 K, which is continuously decreasing with successive measurements. Samples TS2 and TS3 showed PF ~1.25 µW/K^2^ cm and ~1 µW/K^2^ cm, respectively, above 700 K, but they are stable over repeated measurements. Due to the reproducibility of *S* and *ρ*, ODP sintered samples showed reproducible PF. Among all the ODP sintered samples, ODP2 has the highest *PF* ~2.75 µW/K^2^ cm, above 700 K, because of moderate *S* and *ρ*. However, lower *S* and very high *ρ* for samples ODP1 and ODP3, respectively, have an adverse effect on each other, resulting in a comparatively lower *PF* ~1.75 µW/K^2^ cm and ~2.25 µW/K^2^ cm, respectively.

**Figure 7:**
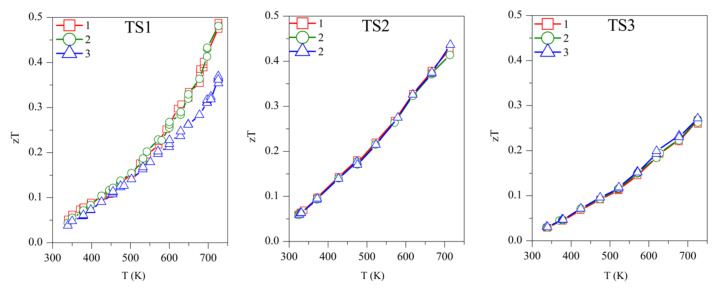

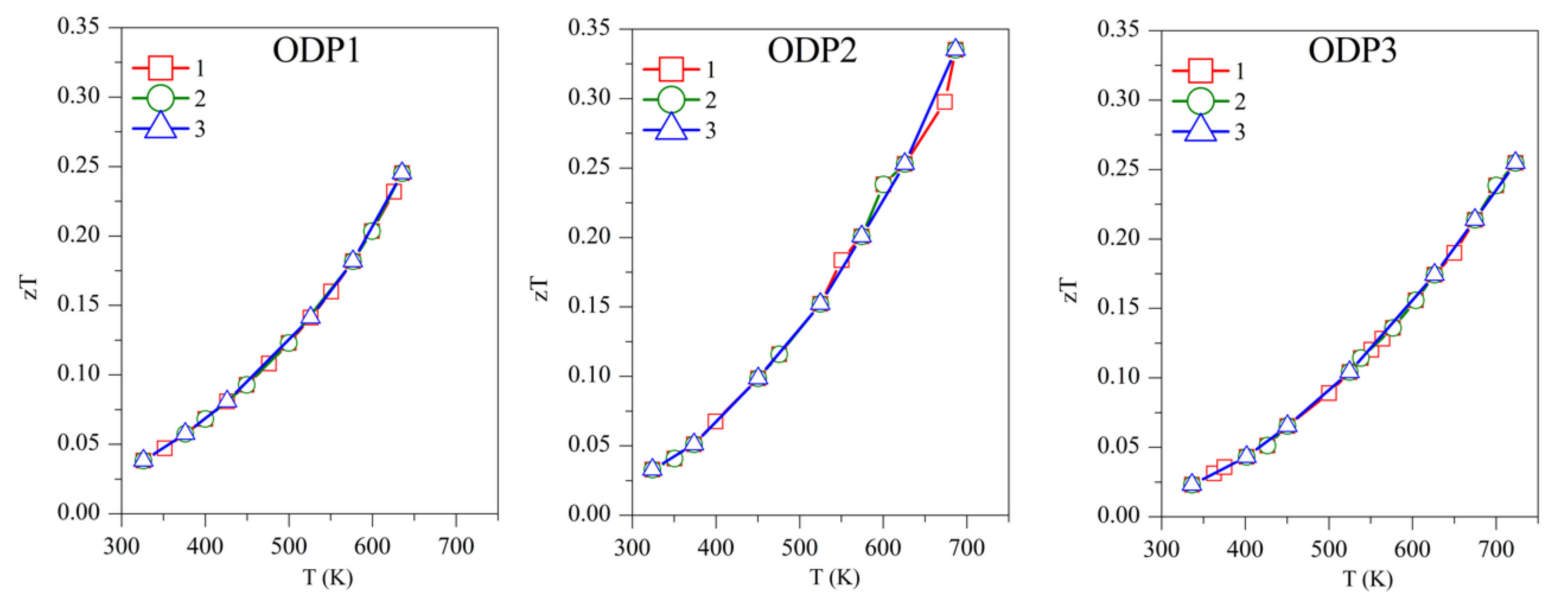
The repeated figure of merit (*zT*) for TS and ODP samples. In the figure, markers 1 (red star), 2 (green circles), and 3 (blue triangles) represent the first, second, and third measurement cycles, respectively.

Concerning the figure of merit, samples TS1, TS2, and TS3 show *zT* ~0.50, 0.45, and 0.27, respectively, above 700 K (Figure 7). Although TS1 has the highest *zT*, it is not stable and *zT* decreases with subsequent measurements. Among the stable TS samples, TS2 has the highest *zT* of ~*0.45*. The competitively high *zT* of TS2 is supported by its lower *κ* and *ρ*. ODP sintered samples present *zT* ranging from 0.25–0.34, around 700 K. The ODP2 sample has the highest *zT* of ~0.34, around 700 K, thanks to the low thermal conductivity throughout the measured temperature span. It is evident from the discussions above that the comparatively higher *zT* of the TS samples is supported by their lower density (high porosity), which blocked the thermal transport effectively. Impotently, the many folds reduction of *κ* due to the pores does not show a significant increase in the resistivity of the TS samples. Furthermore, the porous CTS samples also showed reproducible results during many measurements cycles. Total elimination of secondary phase oxides is not easy due to the low partial pressure of their formations, but a small amount of tin oxides does not seem to affect the TE properties during repeated TE measurements.

## 4. Conclusions

The present work shows that porosity leads to a significant suppression of thermal conductivity, and CTS pellets can be stabilized with different fractions of porosity. The low density or TS samples (*κ* ~0.3–0.2 W/mK) showed ~3 times lower thermal conductivity than the high density or ODP sintered samples (*κ* ~0.8–0.6 W/mK). However, we did not observe any significant difference in electrical transport properties between the TS and ODP samples. The best performing stable TS samples present *zT*~0.45, whereas the best performing ODP sample showed *zT* of ~0.34, around 700 K, a result clearly due to the ultra-low thermal conductivity of the traditionally sintered, porous samples.

Due to the low partial pressure of tin oxides formation, it is difficult to produce pure CTS and similar chalcogenides. However, the continuous evolution of secondary phase oxides can be eliminated by using a highly controlled environment, and a small fraction of secondary phase oxides seem to have little effect on the TE properties and stability of the samples. The results presented in this work give a general overview of the effects of different experimental conditions and porosity on the stability and TE performance of CTS samples. Similar considerations should hold for other Cu-Sn/Fe-S/Se-based systems, chalcogenides, colusites, and chalcopyrite, etc., used for various applications, ranging from photovoltaics to thermoelectricity and LED production.

## Figures and Tables

**Table 1:** Quantitative phase and average crystallite size before and after TE measurements, measured densities, and relative densities.

Sample	Weight (±1%)					Average Domain Size (±10 nm)	Measured Density (±0.1 g/cm^3^)	Relative Density (%)
	CTS	WC	SnO_2_	SnO	SnS			
TS1 before TE measurements	96	2	2	-	-	40	3.52	74.7
TS1 after TE measurements	88	2	8	-	2	75		
TS2 before TE measurements	98	2	-	-	-	75	3.48	73.8
TS2 after TE measurements	89	2	8	1	-	75		
TS3 before TE measurements	98	2	-	-	-	70	3.68	78.1
TS3 after TE measurements	87	2	10	1	-	70		
ODP1 before TE measurements	100	-	-	-	-	42	4.26	90.4
ODP1 after TE measurements	100	-	-	-	-	42		
ODP2 before TE measurements	100	-	-	-	-	54	4.36	92.5
ODP2 after TE measurements	99	1	-	-	-	54		
ODP3 before TE measurements	98	1	1	-	-	68	4.43	94.0
ODP3 after TE measurements	91	1	8	-	-	68		

**Table 2:** Atomic fraction for samples TS1, TS2, and TS3 estimated from the TEM-EDX analysis (corresponding EDX spectra are shown in Figure S4).

Sample Name	Atomic Fraction (±1%)			
	Cu	Sn	S	O
TS1	15.11	26.40	14.92	43.55
TS2	40.09	16.64	41.55	1.7
TS3	30.31	15.43	45.96	3.2

**Table 3:** Atomic fraction of samples ODP1, ODP2, and ODP3 samples, estimated using SEM-EDX analysis (corresponding EDX spectra are shown in Figure S6).

Sample Name	Atomic Fraction (±1%)			
	Cu	Sn	S	O
ODP1	16.90	32.03	46.92	4.05
ODP2	15.46	30.13	45.11	9.27
ODP3	16.82	31.68	49.21	2.29

## Data Availability

The data presented in this study are available on request from the corresponding author.

## Supplementary Materials

The following supporting information can be downloaded at www.mdpi.com/1996-1944/15/3/712/s1. Figure S1: Images of as-sintered ODP samples. Figure S2: Rietveld refinement data before and after many Seebeck and resistivity measurements cycles for all the samples. Figure S3: Morphological images of SPS sintered samples. Figure S4: TEM-EDX, morphological images, and SAED on TE samples. Figure S5: SEM-EDX data for chemical maps collected on ODP samples. Figure S6: Various SEM-EDX data collected on ODP samples. Figure S7: Specific heat capacity (*C_P_*) measurements on ODP samples.
