# Supplementary material for: Effects of Preparation Procedures and Porosity on Thermoelectric Bulk Samples of Cu2SnS3 (CTS)
Source: Materials (Basel). 2022 Jan 18;15(3):712. doi: 10.3390/ma15030712 (PMC8836522; doi:10.3390/ma15030712)
Supplement: Supplementary file 1 [file materials-15-00712-s001.zip › materials-1529756-supplementary.pdf]

Article

Supporting information "Effects of preparation procedures and porosity on thermoelectric bulk samples of Cu_2SnS_3 (CTS)"

Ketan Lohani [1], Carlo Fanciulli [2], and Paolo Scardi [1,*]

[1] Department of Civil, Environmental & Mechanical Engineering, University of Trento, Via Mesiano 77, 38123, Trento, Italy; ketan.lohani@unitn.it
[2] National Research Council of Italy-Institute of Condensed Matter Chemistry and Technologies for Energy (CNR-ICMATE), Lecco Unit, Via Previati 1/E, 23900, Lecco, Italy; carlo.fanciulli@cnr.it
[*] Corresponding author: Paolo.Scardi@unitn.it

Images of as-sintered ODP samples.

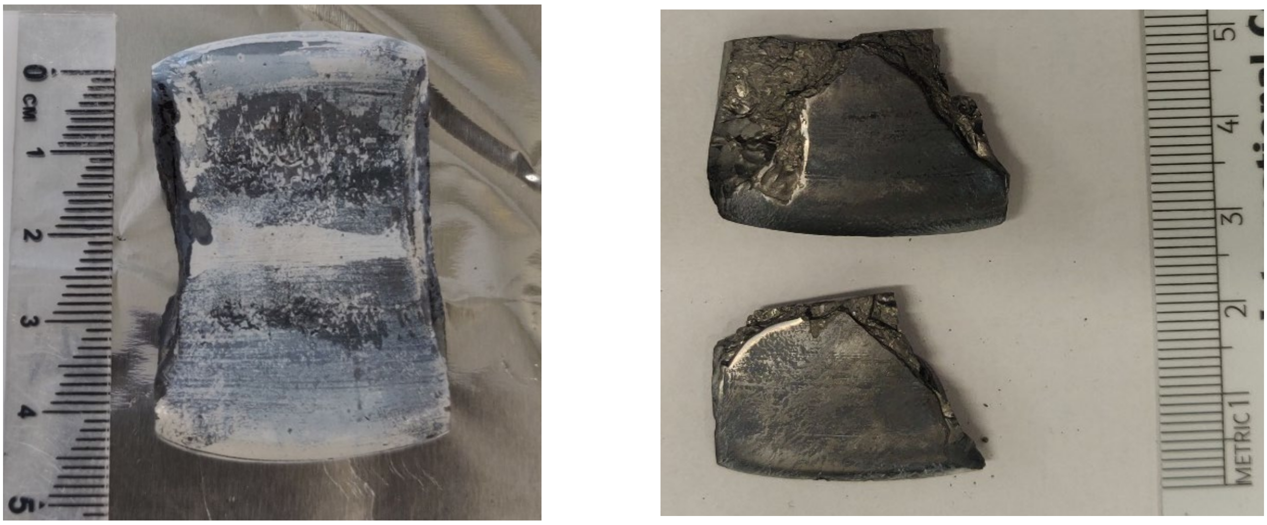

Figure S1. Open die pressed (ODP) sintered CTS samples.

Rietveld refinement was performed on XRD patterns before and after repeated Seebeck and resistivity measurement cycles. The Whole Powder Pattern Modelling (WPPM) method as implemented in Topas 7 software was used for the Rietveld refinement, which considers a log-normal distribution of grains.

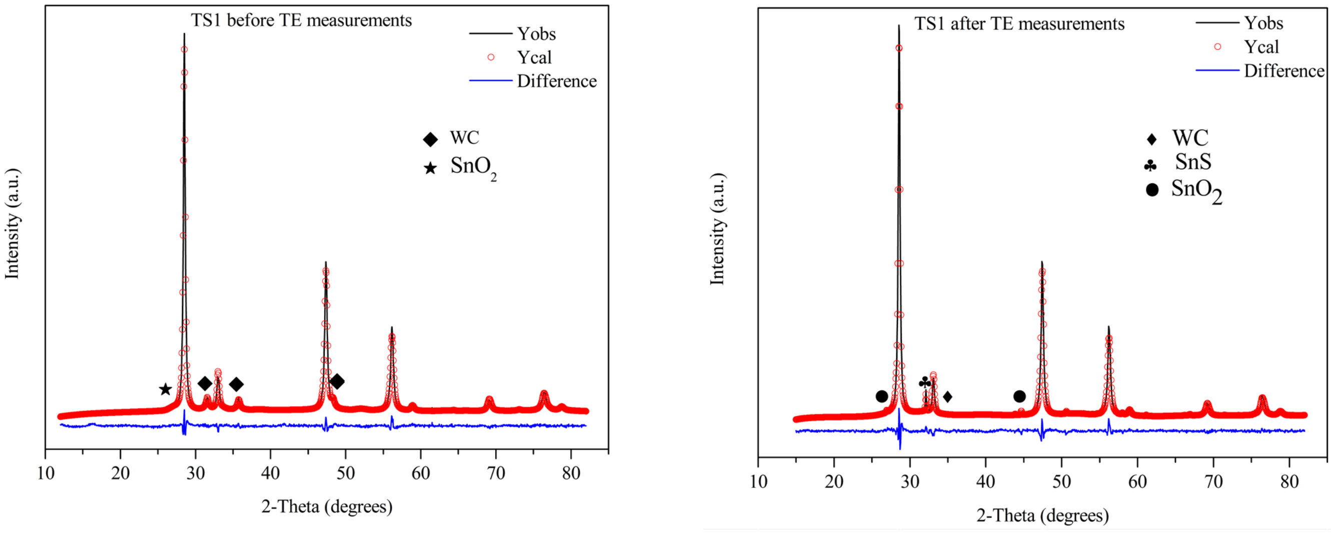

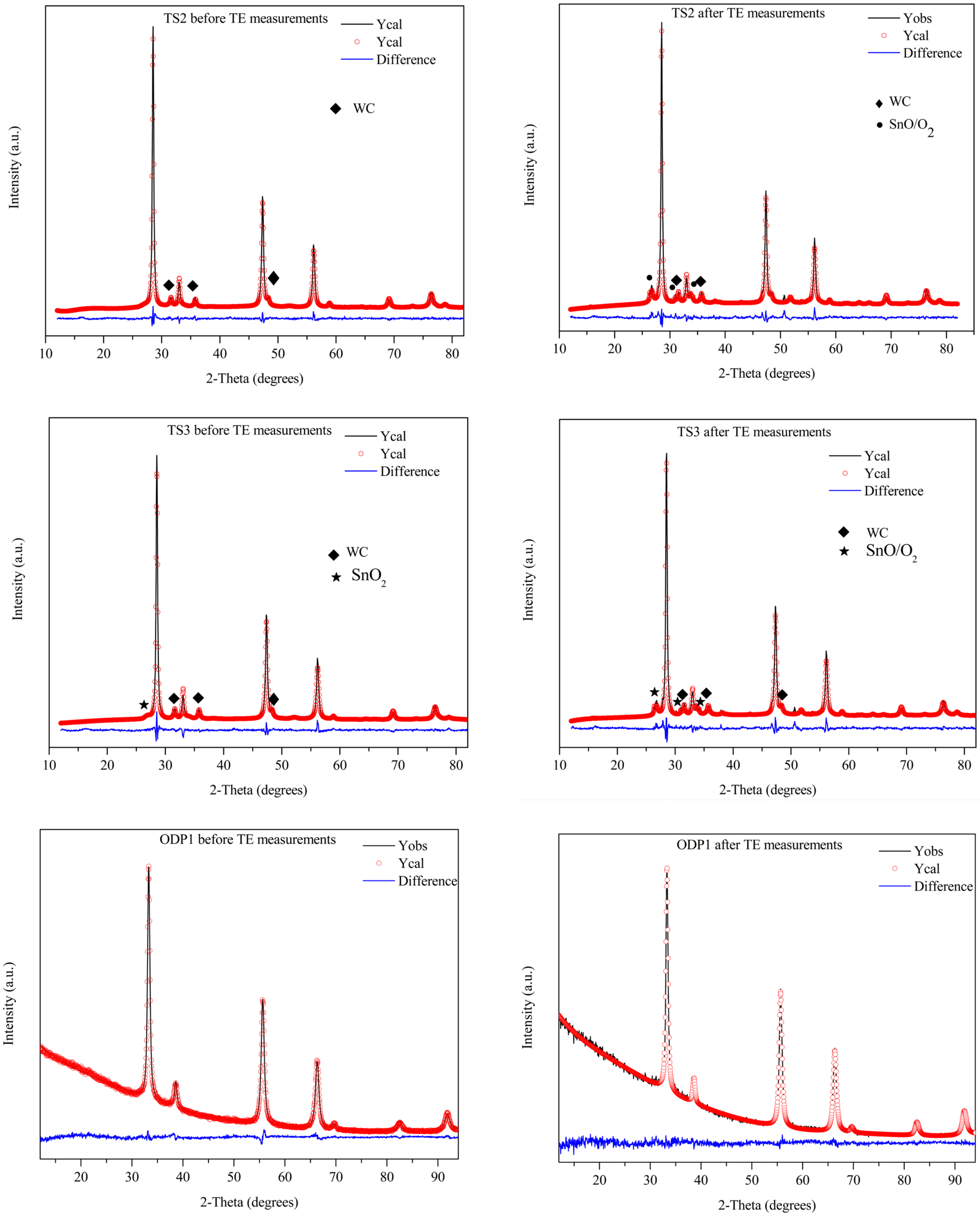
TS2 before TE measurements
Ycal
Ycal
Difference
WC
Intensity (a.u.)
2-Theta (degrees)
TS2 after TE measurements
Yobs
Ycal
Difference
WC
SnO/O2
Intensity (a.u.)
2-Theta (degrees)
TS3 before TE measurements
Ycal
Ycal
Difference
WC
SnO2
Intensity (a.u.)
2-Theta (degrees)
TS3 after TE measurements
Ycal
Ycal
Difference
WC
SnO/O2
Intensity (a.u.)
2-Theta (degrees)
ODP1 before TE measurements
Yobs
Ycal
Difference
Intensity (a.u.)
2-Theta (degrees)
ODP1 after TE measurements
Yobs
Ycal
Difference
Intensity (a.u.)
2-Theta (degrees)

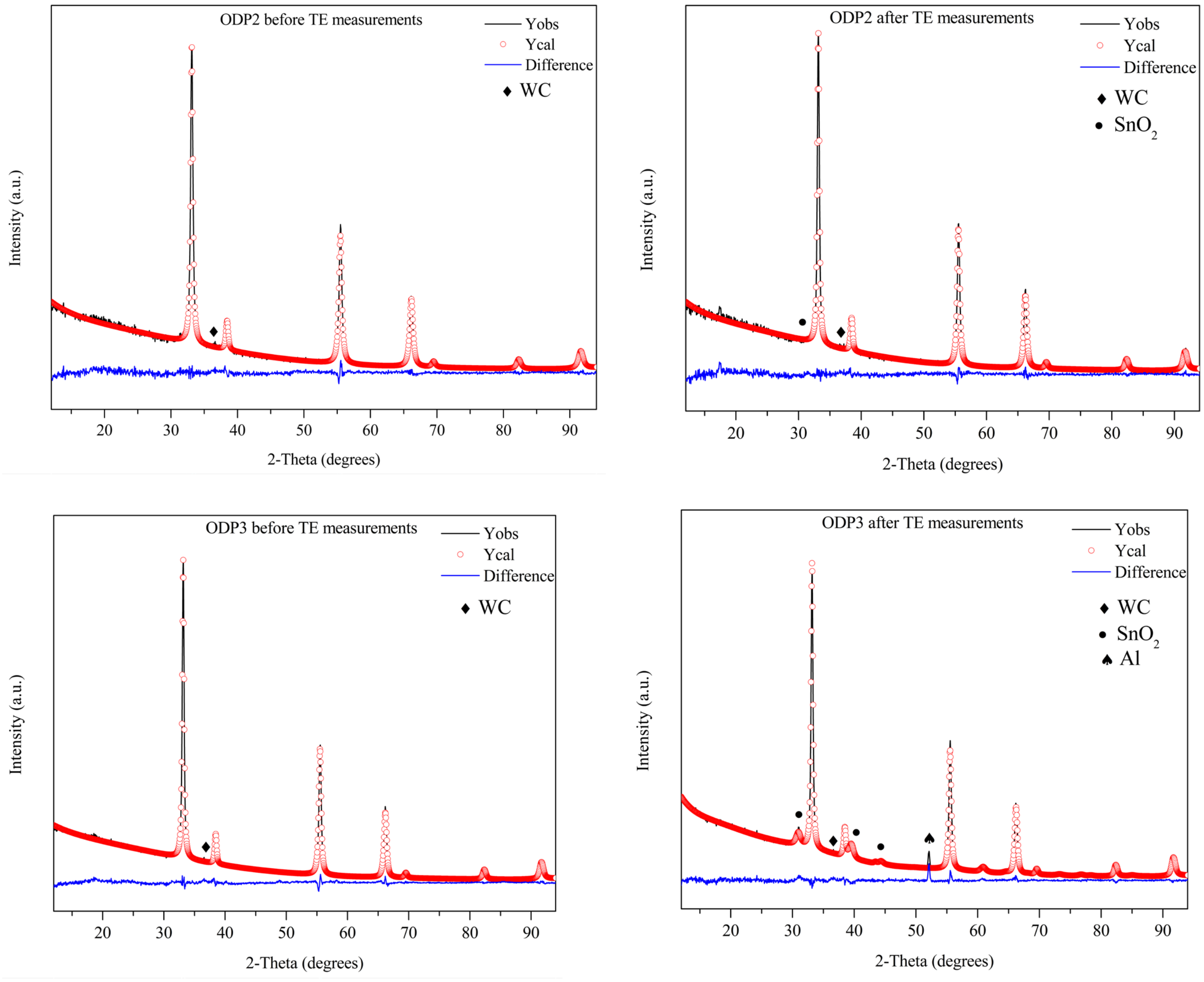

Figure S2. The SAED for all samples shows similar 3 high-intensity Debye-Scherrer rings, representing the fingerprint planes for cubic CTS, (1 1 1), (2 2 0), and (3 1 1), respectively, as observed in the XRD patterns.

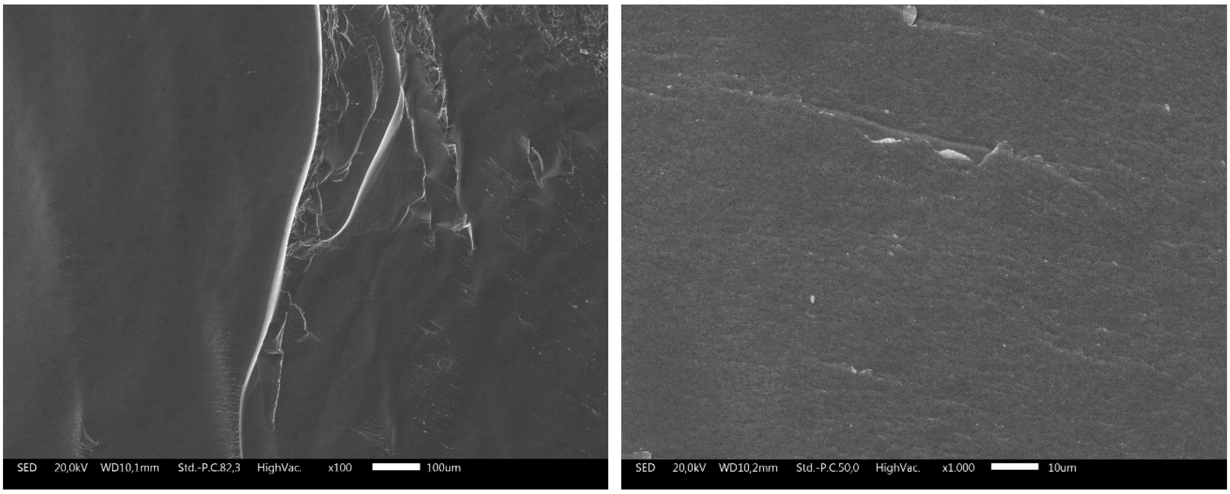

Figure S3. Morphological image of SPS Sintered CTS Sample at different magnifications.

The SAED for all the samples shows similar 3 high-intensity Debye-Scherrer rings, representing the fingerprint planes for cubic CTS, (1 1 1), (2 2 0), and (3 1 1), respectively, as observed in the XRD patterns.

The micrographs collected on sample TS1 is shown in Figure S4 (right bottom inset) revealed numerous small SnO_2 grains surrounding the bigger (CTS) grains. In the cases of samples TS2 and TS3, these grains were not observed. However, we observed some variations in the chemistry.

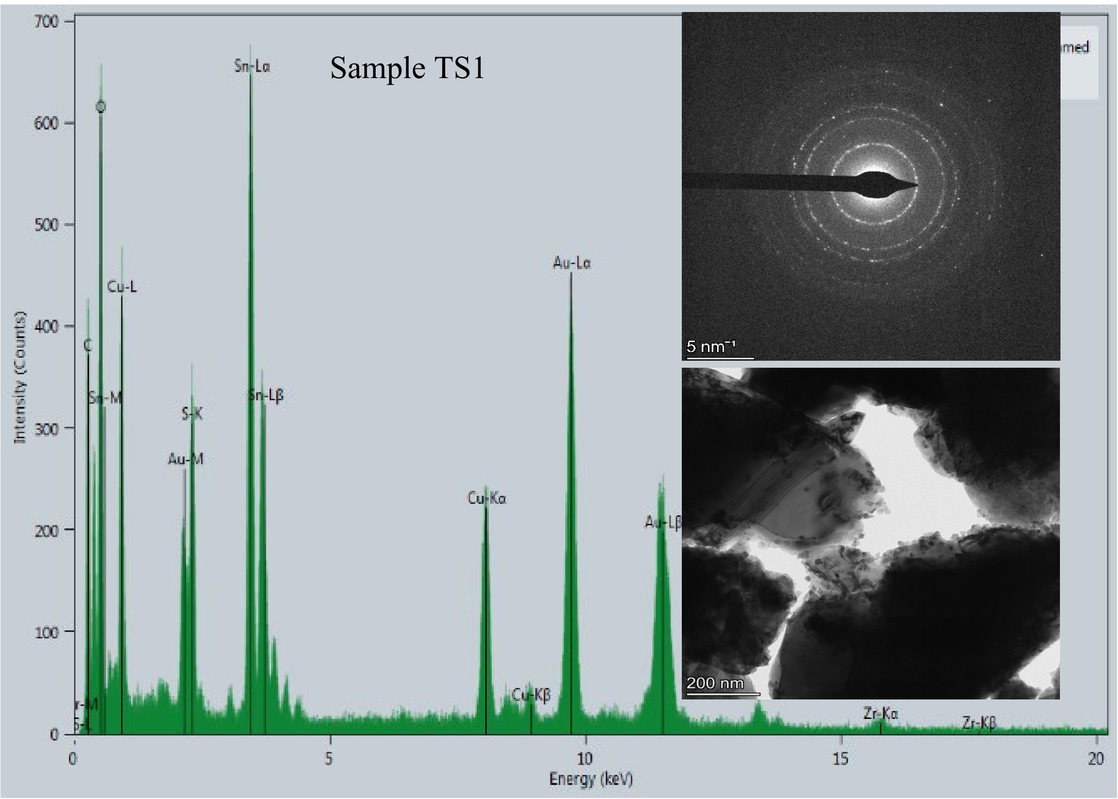

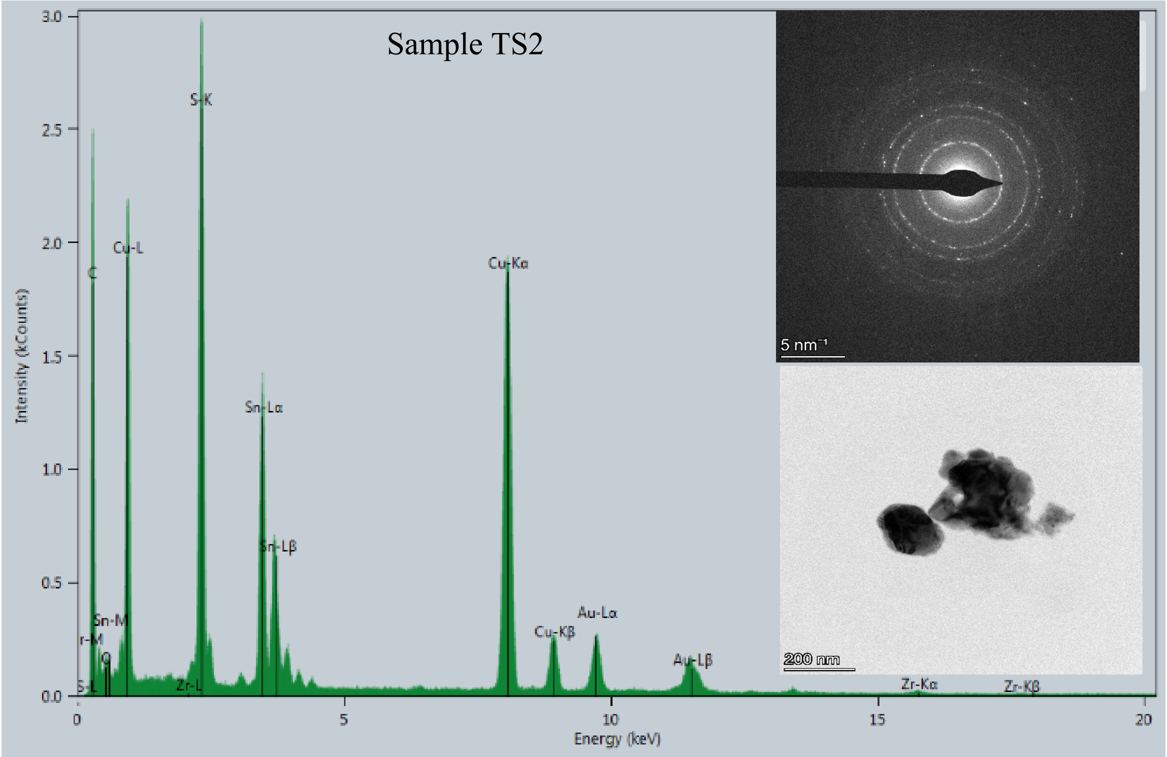

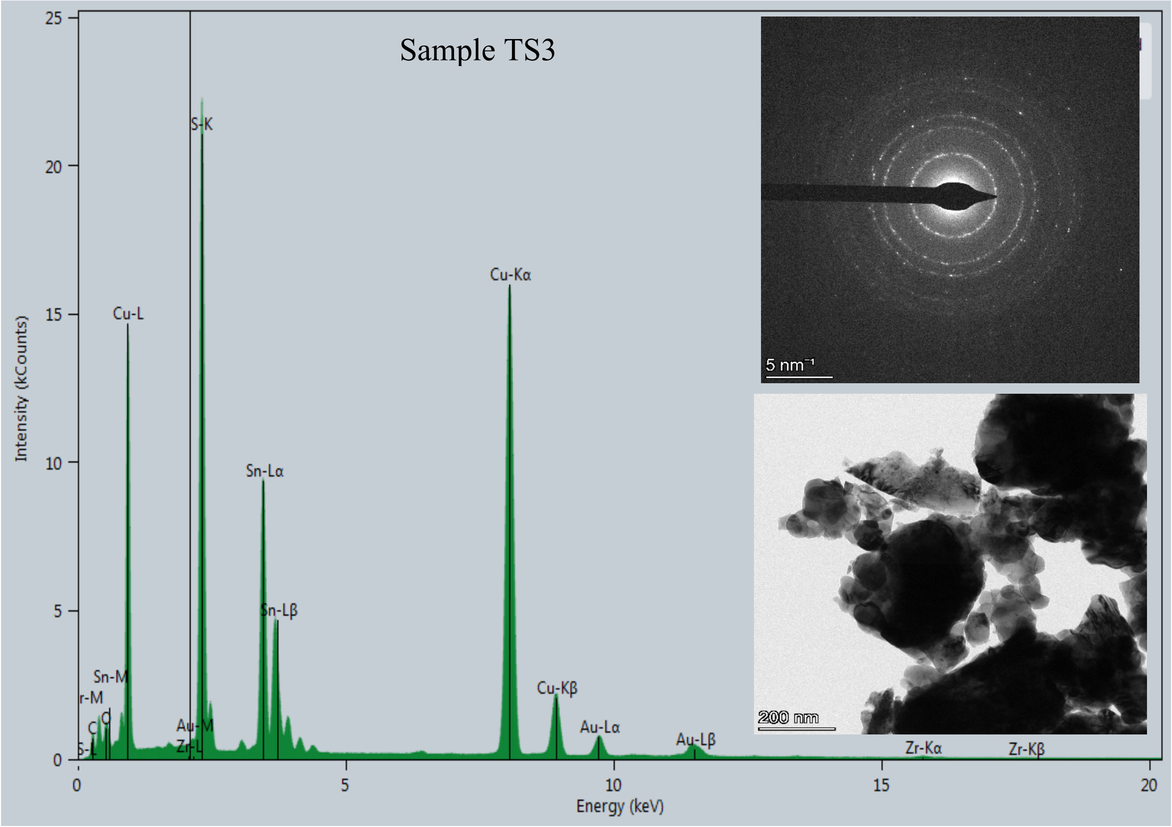

Figure S4. EDX-spectra, SAED, and TEM micrographs for samples TS1, TS2, and TS3, respectively.

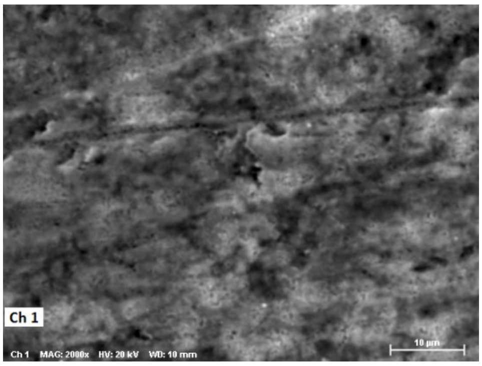

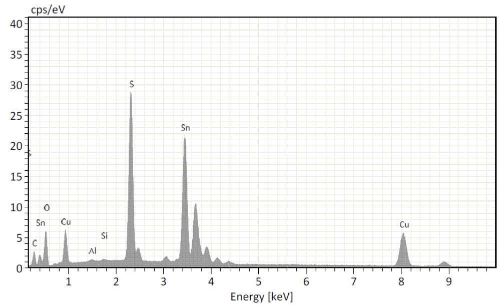

Element	At. No.	Netto	Mass [%]	Mass Norm. [%]	Atom [%]	abs. error [%] (1 sigma)	rel. error [%] (1 sigma)
Sulfur	16	135624	18.02	19.83	41.49	0.67	3.73
Copper	29	46729	24.44	26.89	28.39	0.69	2.82
Tin	50	200451	48.42	53.28	30.12	1.45	3.00
		Sum	90.87	100.00	100.00		

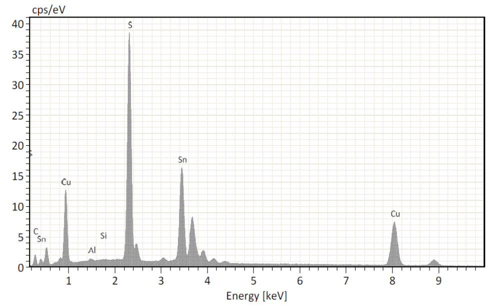

Element	At. No.	Netto	Mass [%]	Mass Norm. [%]	Atom [%]	abs. error [%] (1 sigma)	rel. error [%] (1 sigma)
Sulfur	16	182434	23.74	25.69	47.75	0.88	3.69
Copper	29	61688	31.70	34.31	32.17	0.88	2.79
Tin	50	151456	36.96	40.00	20.08	1.12	3.02
		Sum	92.41	100.00	100.00		

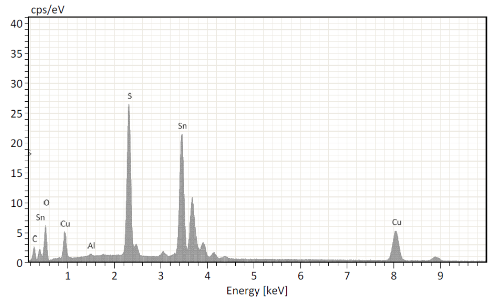

Element	At. No.	Netto	Mass [%]	Mass Norm. [%]	Atom [%]	abs. error [%] (1 sigma)	rel. error [%] (1 sigma)
Sulfur	16	124245	17.13	18.66	39.99	0.64	3.74
Copper	29	43491	23.62	25.73	27.82	0.67	2.83
Tin	50	200480	51.07	55.62	32.19	1.53	3.00
		Sum	91.83	100.00	100.00		

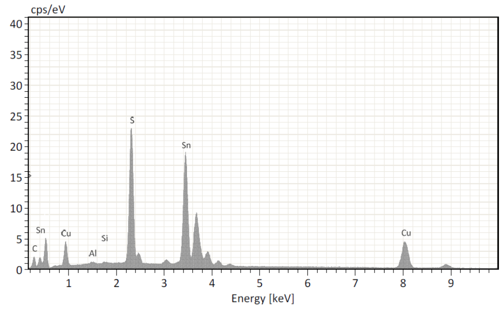

Element	At. No.	Netto	Mass [%]	Mass Norm. [%]	Atom [%]	abs. error [%] (1 sigma)	rel. error [%] (1 sigma)
Sulfur	16	107760	16.92	18.77	40.08	0.63	3.74
Copper	29	37322	23.49	26.06	28.08	0.67	2.84
Tin	50	171768	49.74	55.18	31.83	1.49	3.00
		Sum	90.14	100.00	100.00		

Figure S5. Corresponding SEM-EDX data on chemical maps shown in Figure 3 for individual elements (Cu, Sn, and S) and simultaneously for all elements.

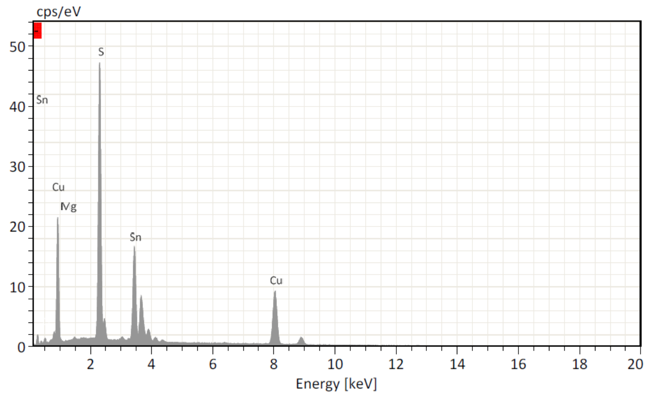

Element	At. No.	Netto	Mass [%]	Mass Norm. [%]	Atom [%]	abs. error [%] (1 sigma)	rel. error [%] (1 sigma)
Oxygen	8	1403	1.11	1.19	4.16	0.25	22.94
Sulfur	16	220367	25.02	26.81	46.92	0.92	3.68
Copper	29	76078	33.84	36.26	32.03	0.94	2.77
Tin	50	148533	33.35	35.74	16.90	1.01	3.03
		Sum	93.32	100.00	100.00		

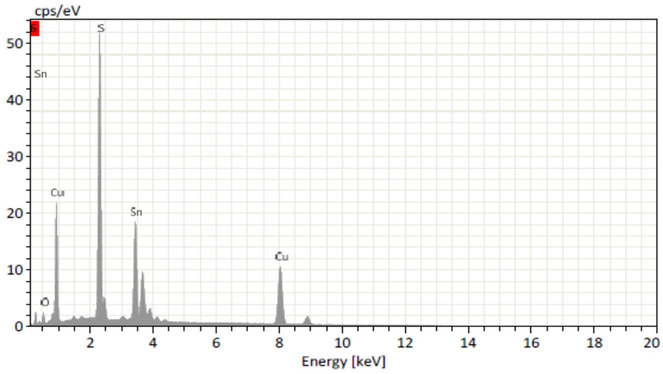

Element	At. No.	Netto	Mass [%]	Mass Norm. [%]	Atom [%]	abs. error [%] (1 sigma)	rel. error [%] (1 sigma)
Oxygen	8	3852	2.64	2.77	9.27	0.46	17.39
Sulfur	16	242471	25.72	27.05	45.11	0.95	3.68
Copper	29	87490	34.04	35.80	30.13	0.94	2.77
Tin	50	168949	32.69	34.38	15.49	0.99	3.03
		Sum	95.08	100.00	100.00		

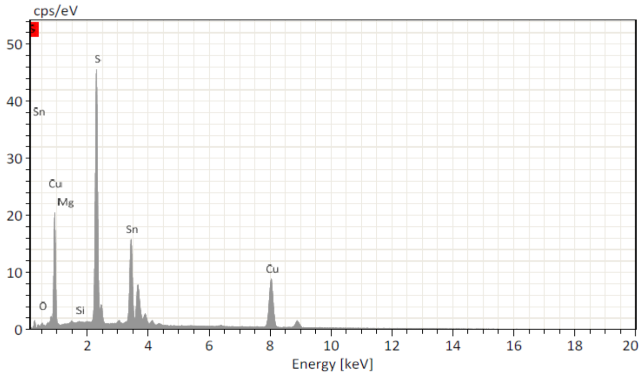

Element	At. No.	Netto	Mass [%]	Mass Norm. [%]	Atom [%]	abs. error [%] (1 sigma)	rel. error [%] (1 sigma)
Oxygen	8	730	0.62	0.65	2.29	0.18	28.84
Sulfur	16	212711	26.74	28.06	49.21	0.98	3.68
Copper	29	72644	34.11	35.79	31.68	0.95	2.77
Tin	50	142692	33.84	35.51	16.82	1.02	3.03
		Sum	95.32	100.00	100.00		

Figure S6. SEM-EDX on ODP1, ODP2, and ODP3 samples.

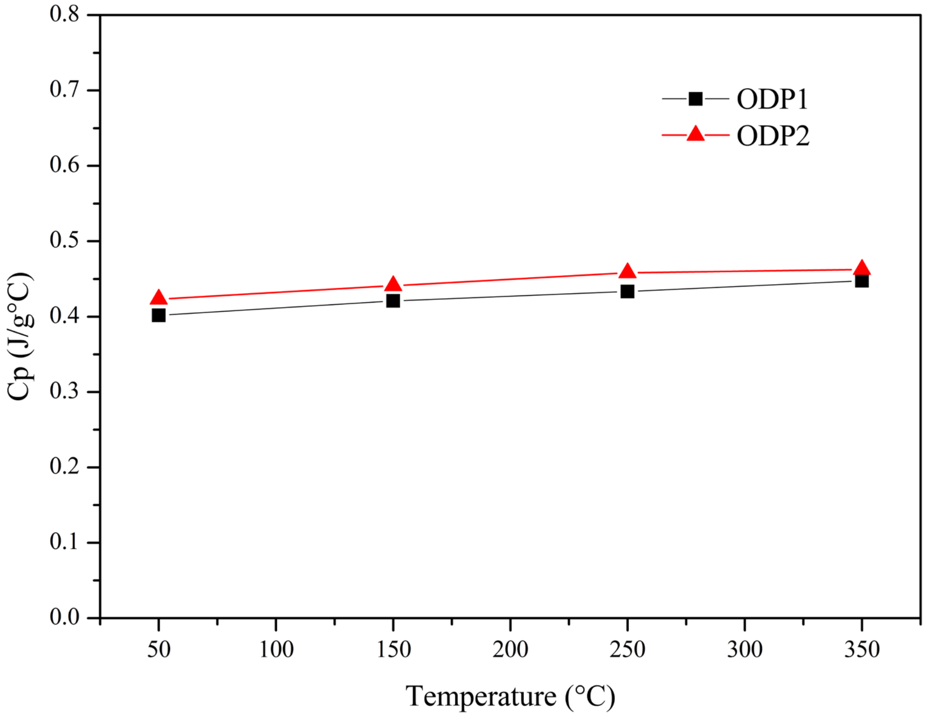

Figure S7. Specific heat capacity (C_P) measurements on ODP sintered samples.
